# A Novel Hyperchaotic 2D-SFCF with Simple Structure and Its Application in Image Encryption

**DOI:** 10.3390/e24091266

**Published:** 2022-09-09

**Authors:** Yongsheng Hu, Han Wu, Luoyu Zhou

**Affiliations:** 1 School of Information Engineering, Binzhou University, Binzhou 256603, China; 2 School of Electronics and Information, Yangtze University, Jingzhou 434023, China

**Keywords:** chaotic image encryption, chaos theory, hyperchaotic, cryptography

## Abstract

In this paper, a novel image encryption algorithm is proposed based on hyperchaotic two-dimensional sin-fractional-cos-fractional (2D-SFCF), called sin-fractional-cos-fractional image-encryption (SFCF-IE). The 2D-SFCF is constructed from two one-dimensional cosine fractional (1-DCFs), and it has a more complex chaotic behavior with a larger parameter space than one-dimensional chaotic systems. Compared with the two-dimensional (2D) chaotic system, the 2D-SFCF has a simple structure, and the parameter space in the chaotic state is continuous, which is beneficial to generating the keystream in the cryptosystem. Therefore, in the novel image encryption algorithm, we use the 2D-SFCF to generate the keystream of the cryptosystem. The encryption algorithm is a process of scrambling and diffusion. Different from common diffusion methods, the diffusion starting position of the SFCF-IE is randomly generated, enhancing the algorithm’s security. Simulation experiments show that the image encrypted by this algorithm has better distribution characteristics and can resist common attack methods.

## 1. Introduction

Images are the basis of human vision. Since digital images are vivid and intuitive, the most important means for people to obtain information is to obtain information from images in daily life [1,2]. With the accelerated development of the Internet, the study of ensuring the secure transmission of images in the network has become one of the hot issues [3,4,5]. Compared with text information, the image has a two-dimensional structure and the adjacent pixels have a strong correlation. Therefore, text information encryption algorithms are unsuitable for image encryption, such as 3DES and AES [6].

Many image protection methods have been proposed, such as image hiding technology, image watermarking technology, and image encryption technology [7,8,9,10,11]. Among these image protection techniques, image encryption is the most direct way, which converts the original plaintext information into a noisy image. The image encryption algorithm consists of two steps, scrambling and diffusion. The scrambling part is to change the position of the original plaintext pixel value, and the diffusion algorithm is to change the value of the original plaintext pixel [12,13].

Because the chaotic system is highly sensitive to the change of the initial value, a large number of excellent pseudorandom sequences can be generated by the chaotic system, which is consistent with the keystream required for scrambling and diffusion of image encryption. Therefore, the image encryption algorithm combined with chaos theory has gradually become the main research method for image encryption algorithms [14,15,16,17,18,19,20]. In Ding’s algorithm, two chaotic systems are used to generate the keystream, fractional-order Henon is used for shuffling, and the keystream generated by the 4D hyperchaotic system is used in the diffusion stage [15]. Li et al. proposed a fractional-order chaotic system and simulated it on the DSP platform, then proposed a new image encryption algorithm using this fractional-order chaotic system [17]. Chai et al. used a Four-wing hyperchaotic system to generate a keystream and used DNA technology in image encryption. The experimental results verified that the algorithm has good performance. However, the efficiency of DNA decoding and encoding operations in computer simulations is slow [20].

For the chaotic image encryption algorithm, the security is mainly related to the performance of the chaotic system. Chaotic systems are divided into low-dimensional and high-dimensional chaotic systems [21,22,23]. The structure of low-dimensional chaotic systems is simple and easy to generate, and many image encryption methods based on low-dimensional chaos have been proposed [24,25,26]. However, low-dimensional systems have the disadvantages of small parameter space and no complex dynamic behavior, and their trajectories are easily estimated on computer platforms with limited precision [27,28]. Compared with low-dimensional chaotic systems, high-dimensional chaotic systems have larger parameter spaces, more complex structures, and better dynamic behavior. Many high-dimensional chaos-based image encryption methods have been proposed today [29,30,31]. However, due to the complex structure of the high-dimensional mixed-degree system, the efficiency of generating the keystream is slow, and it is difficult to achieve in industrial production.

To balance the disadvantages and advantages of high-dimensional chaotic systems and low-dimensional chaotic systems. We propose a new two-dimensional chaotic system called 2D-SFCF. The 2D-SFCF evolved from two 1-DCFs [32]. The 2D-SFCF has a larger parameter space and better dynamic behavior than one-dimensional chaotic systems. The 2D-SFCF is a hyperchaotic system. Compared with chaotic behavior, hyperchaotic behavior is a more complex state. Compared with high-dimensional chaotic systems, the 2D-SFCF has a simpler structure, and this simple structure can also evolve complex dynamic behavior, and the speed of the 2D-SFCF generating keystream is fast.

Based on the excellent performance of the 2D-SFCF, we designed a new image encryption algorithm called SFCF-IE. The SFCF-IE is a scramble-to-diffusion process. A hash function generates the key of the SFCF-IE, which is converted into the initial value and parameters of the 2D-SFCF to generate the keystream required for scrambling and diffusion. The scrambling adopts a random scrambling method. In the diffusion algorithm, the starting position of the diffusion is randomly generated, not the starting position of the image pixel value. This diffusion method enhances the security of the encryption algorithm. In addition, the SFCF-IE only needs one round of encryption to achieve the security required by the cryptosystem, and the image encrypted by the SFCF-IE has good distribution characteristics.

## 2. 2D-SFCF

In this paper, a new 2D-SFCF is proposed, mathematical expression of the 2D-SFCF is shown in Equation (1),

(1) $$\left\{\begin{array}{c} x_{i+1}=\cos\left(\frac{\pi \alpha }{{y_{i}}^{\beta }}\right)\\ y_{i+1}=\sin\left(\frac{\pi \alpha }{{x_{i}}^{\beta }}\right) \end{array}\right.$$

There are two inputs and two outputs in the 2D-SFCF. α and β are the control parameters of the 2D-SFCF, $\alpha \in R^{+}$ and $\beta \in N^{+}$. $x_{i}$ and $y_{i}$ are the iterative sequence, $x_{i}\in \left(-1,1\right)$ and $y_{i}\in \left(-1,1\right)$.

2D-SFCF is evolved from 1-DCF [32], and its expression is shown in Equation (2),

(2) $$x_{n+1}=\cos\left(\frac{\alpha }{{x_{n}}^{\beta }}\right).$$

Compared with the 1-DCF, the dynamic behavior of the 2D-SFCF is more complex and the parameter space in chaotic state is larger.

### 2.1. Attractor

**Figure 1:**
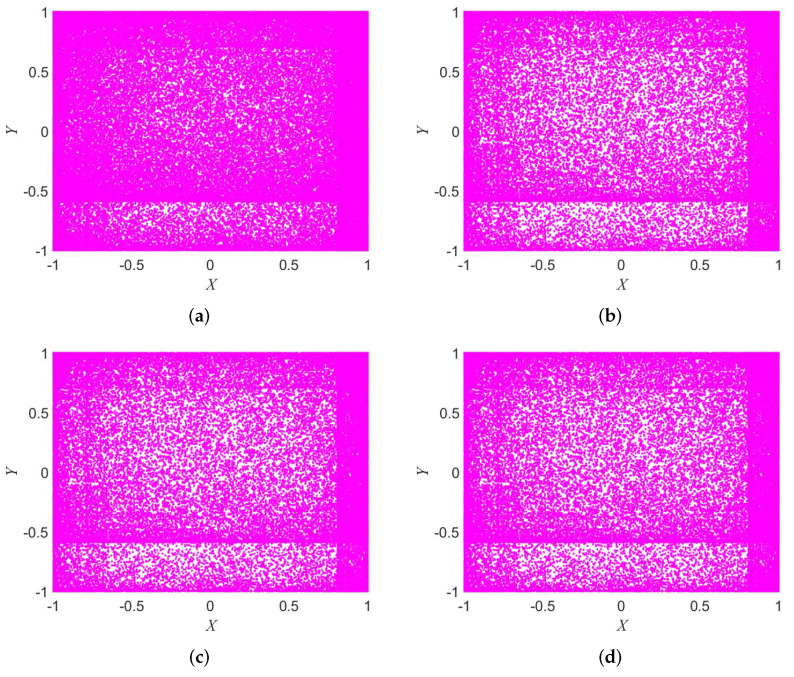
Attractor of 2D-SFCF. (**a**) α=1.8,β=2. (**b**) α=3,β=4.2. (**c**) α=5,β=9.7. (**d**) α=8,β=9.7.

The attractor indicates the ergodicity of the system. If the nonlinear dynamical system has good ergodicity, its attractor distribution will fill the entire phase space. The nonlinear dynamic system with strong ergodicity is more suitable for the cryptographic system. The cryptographic system requires random keys and this is not easy to predict. The attractors of the 2D-SFCF are shown in Figure 1 in different parameter. The initial value is set to $x_{0}=0.78345533315$ and $y_{0}=0.97443453789$.

It can be seen from Figure 1 that the 2D-SFCF has good ergodicity, and its attractors are evenly distributed in the phase space, so it can be shown that the chaotic sequence generated by the 2D-SFCF has good randomness.

### 2.2. NIST Statistical Test Suite

The National Institute of Standards and Technology (NIST) is an important tool for counting the randomness of a sequence. Given the significance level, when the test value is greater than the significance level, it indicates that the sequence passes the test and is random. In this section, given the significance level γ=0.01, the NIST test results of the 2D-SFCF are shown in Table 1, where the initial values of the 2D-SFCF are $x_{0}=0.78345533315$ and $y_{0}=0.97443453789$. The NIST test results show that the sequences generated by the 2D-SFCF have a high degree of randomness. The 2D-SFCF is suitable for cryptosystem to generate keystream.

### 2.3. Lyapunov Exponents

The Lyapunov exponent is recognized as one of the most effective means of judging whether a nonlinear dynamical system is chaotic. The calculation formula of Lyapunov exponents is shown in Equation (3),

(3) $$\lambda =\lim_{T\to +\infty }\frac{1}{T}\sum_{t=0}^{T}|f^{\prime }\left(x_{t}\right)|.$$

**Figure 2:**
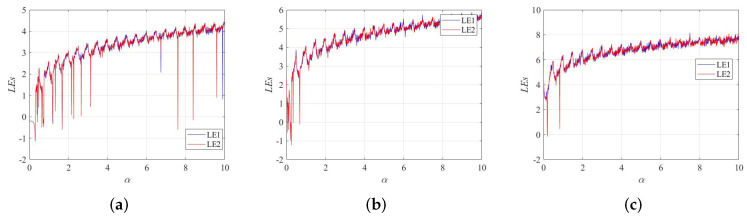

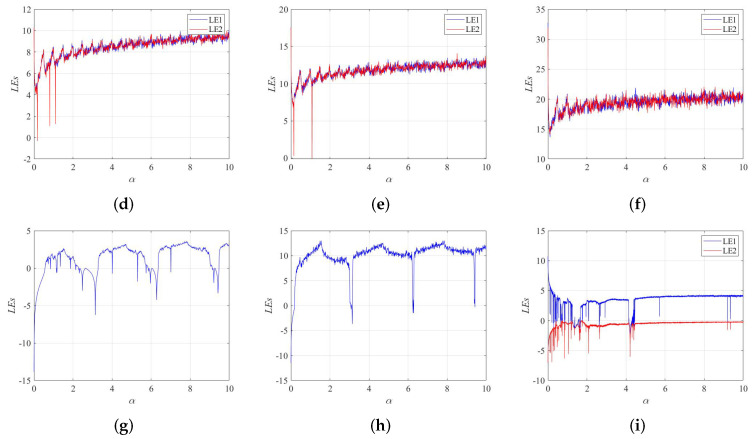
Lyapunov exponents. (**a**) LEs of 2D-SFCF with β=1. (**b**) LEs of 2D-SFCF with β=2. (**c**) LEs of 2D-SFCF with β=4. (**d**) LEs of 2D-SFCF with β=6. (**e**) LEs of 2D-SFCF with β=10. (**f**) LEs of 2D-SFCF with β=20. (**g**) LEs of 1-DCF with β=1. (**h**) LEs of 1-DCF with β=10. (**i**) LEs of 2D-SCMCI.

When the nonlinear dynamical system has more than one positive Lyapunov exponent, it indicates that the system is in a hyperchaotic state. A hyperchaotic state is a more complex dynamical behavior than a chaotic state. The Lyapunov exponents of the 2D-SFCF are shown in Figure 2a–f. Furthermore, it is compared with the Lyapunov exponents of the 1-DCF [32] and the 2D-SCMCI [33], which are shown in Figure 2g–i.

**Figure 3:**
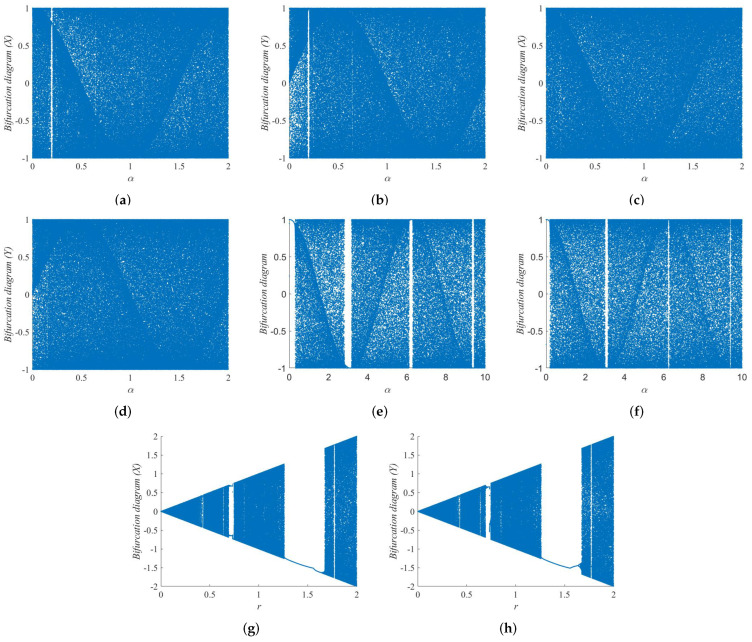
Bifurcation diagram (BD). (**a**) BD of 2D-SFCF with β=4 (X). (**b**) BD of 2D-SFCF with β=4 (Y). (**c**) BD of 2D-SFCF with β=10 (X). (**d**) BD of 2D-SFCF with β=10 (Y). (**e**) BD of 1-DCF with β=4. (**f**) BD of 1-DCF with β=10. (**g**) BD of 2D-SCMCI (X). (**h**) BD of 2D-SCMCI (Y).

Compared with the 1-DCF, the 2D-SFCF has a larger parameter space in the chaotic state. In the same parameter space, the 2D-SFCF exhibits a hyperchaotic state, while the 1-DCF is in a chaotic state or even a periodic state. Compared with the 2D-SCMCI, the parameter space of 2D-SFCF in the chaotic state is continuous, which is beneficial to generating the secret key of the cryptosystem. The comparison results show that the 2D-SFCF has good kinetic behavior. Furthermore, we verify the accuracy of Lyapunov exponents using bifurcation diagrams, the bifurcation diagrams of the 2D-SFCF are shown in Figure 3a–d. The bifurcation diagrams of the 1-DCF are shown in Figure 3e,f. The bifurcation diagrams of the 2D-SCMCI are shown in Figure 3g,h.

## 3. SFCF-IE Algorithm

**Figure 4:**
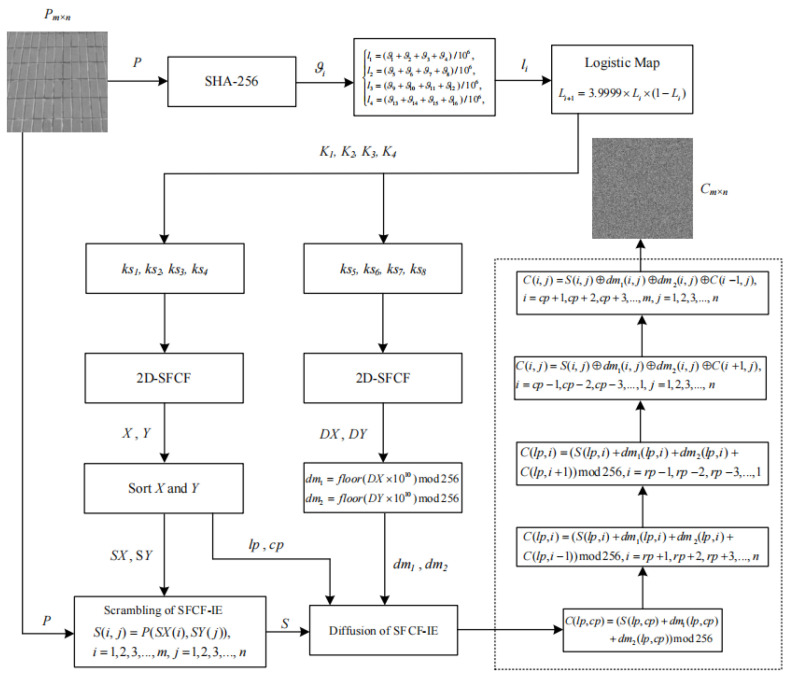
Schematic diagram of SFCF-IE.

The SFCF-IE is divided into three parts, key generation, scrambling, and diffusion. The SFCF-IE is a symmetric cryptosystem, and the decryption process is the reverse process of encryption. The structure of the SFCF-IE is shown in Figure 4.

### 3.1. Function Declaration

(1) Set a key processing function [A,B,C,D]=F(a,b,c,d), the function *F* can be converted a,b,c,d into a new value A,B,C,D by Equation (4),
  (4) $$\left\{\begin{array}{c} A=(a\;\mathrm{mod}\;1)\times 100+1,\\ B=floor(b\times 10^{10})\;\mathrm{mod}\;35+2,\\ C=c\;\mathrm{mod}\;1,\\ D=d\;\mathrm{mod}\;1. \end{array}\right.$$
(2) Set to a keystream generation function [SX,SY]=C(a,b,c,d,N), where *a* is the parameter α of the 2D-SFCF, *b* is the parameter β of the 2D-SFCF, *c* is the initial value $x_{0}$ of the 2D-SFCF, *d* is the initial value $x_{0}$ of the 2D-SFCF, and *N* is the number of iterations. Output sequences are $SX\in M_{1\times N}$ and $SY\in M_{1\times N}$. Note that when generating a chaotic sequence, some initial values of iterations need to be discarded, so that the generated sequence is sufficiently chaotic. Here, the first 100 iteration values are set to be discarded.
(3) Set a sorting function B=Sort(a), which can sort the one-dimensional vector *a* and find the position of the sorted vector in the vector *a*, and the return value is *B*. An example of a sorting function. If $A=\left[\begin{array}{cccccc} 0.2785 & 0.5469 & 0.9575 & 0.9649 & 0.1576 & 0.9706 \end{array}\right]$, and then $B=\left[\begin{array}{cccccc} 4 & 5 & 2 & 3 & 6 & 1 \end{array}\right]$.

### 3.2. Key Generation of SFCF-IE

The key of the SFCF-IE is generated by a hash function. The initial key is a 256-bit binary stream. The initial key is processed so that the initial key becomes the initial value and parameter of the 2D-SFCF. The key generation process is described as follows.

Input: *P* ($P\in M_{m\times n}$)

Step 1: Use SHA-256 to generate the initial key Ψ, which is a 256-bit key, and the input to the hash function is a plaintext image.

Step 2: Every 16 bits in Ψ is converted into a new key $\vartheta_{i}$, which is a decimal, $\vartheta_{i}\in \left[0,2^{16}\right]$, i=1,2,3,…,16.

Step 3: Processing $\vartheta_{i}$, so that $\vartheta_{i}$ can become the initial value of Logistic,

(5) $$\left\{\begin{array}{c} l_{1}=\left(\vartheta_{1}+\vartheta_{2}+\vartheta_{3}+\vartheta_{4}\right)/10^{6},\\ l_{2}=\left(\vartheta_{5}+\vartheta_{6}+\vartheta_{7}+\vartheta_{8}\right)/10^{6},\\ l_{3}=\left(\vartheta_{9}+\vartheta_{10}+\vartheta_{11}+\vartheta_{12}\right)/10^{6},\\ l_{4}=\left(\vartheta_{13}+\vartheta_{14}+\vartheta_{15}+\vartheta_{16}\right)/10^{6}, \end{array}\right.$$

Step 4: $l_{i}$ are the initial value of Logistic, iterate 30 times by Equation (6),

(6) $$\left\{\begin{array}{c} L(j,i+1)=3.9999\times L(j,i)\times (1-L(j,i)),\\ L\left(1,1\right)=l_{1},L\left(2,1\right)=l_{2},L\left(3,1\right)=l_{3},L\left(4,1\right)=l_{4},\\ j=1,2,3,4,i=1,2,3,\ldots ,30. \end{array}\right.$$

Step 5: The new keys are set to $K_{1}=L\left(1,30\right)$, $K_{2}=L\left(2,30\right)$, $K_{3}=L\left(3,30\right)$, and $K_{4}=L\left(4,30\right)$.

Output: $K_{1},K_{2},K_{3}$ and $K_{4}$

### 3.3. Scrambling of SFCF-IE

The scrambling algorithm is described as follows.

Step 1: Process the generated secret key $K_{1},K_{2},K_{3}$, and $K_{4}$, $K_{1},K_{2},K_{3}$. Get the initial values and parameters of the 2D-SFCF.

Step 2: Generate a key stream by 2D-SFCF, $\left[X,Y\right]=C(ks_{1},ks_{2},ks_{3},ks_{4},\max\left(m,n\right))$, and output two sequences, $X\in M_{1\times \max(m,n)}$ and $Y\in M_{1\times \max(m,n)}$.

Step 3: Sort *X* and *Y*, generate a row scrambled matrix SX and a column scrambled matrix SY, where SX=sort(X(1:m)) and SY=sort(Y(1:n)).

Step 4: Scramble the plaintext *P* by Equation (7),

(7) S(i,j)=P(SX(i),SY(j)),i=1,2,3,…,m,j=1,2,3,…,n.

where *S* is the scrambled matrix.

### 3.4. Diffusion of SFCF-IE

Different from the common diffusion algorithm that starts from position (1,1), the starting position of the SFCF-IE diffusion algorithm is determined by the secret key. This design method increases the diversity of the algorithm and increases the difficulty of cracking. The diffusion algorithm is described as follow.

Input: *S* ($S\in M_{m\times n}$)

Step 1: The starting positions lp and cp of the diffusion is generated by the sequence *X* and *Y*,

(8) $$\begin{array}{c} lp=\mathrm{floor}(X\left(\mathrm{floor}\left(m/2\right)+1\right)\times 10^{10}\;\mathrm{mod}\;\left(m-1\right)+1)\\ cp=\mathrm{floor}(Y\left(\mathrm{floor}\left(n/2\right)+1\right)\times 10^{10}\;\mathrm{mod}\;\left(n-1\right)+1) \end{array}.$$

Step 2: The matrixs $dm_{1},dm_{2}$ required for diffusion are generated by Equation (9),

(9) $$\begin{array}{c} \begin{array}{c} \left[ks_{5},ks_{6},ks_{7},ks_{8}\right]=F\left(K_{1},K_{2},K_{3}+K_{4},K_{4}-K_{3}\right)\\ \left[DX,DY\right]=C\left(ks_{5},ks_{6},ks_{7},ks_{8},m\times n\right)\\ dm_{1}=floor\left(DX\times 10^{10}\right)\;\mathrm{mod}\;256\\ dm_{2}=floor\left(DY\times 10^{10}\right)\;\mathrm{mod}\;256 \end{array} \end{array}$$

Convert $dm_{1},dm_{2}$ to two matrixs, $dm_{1}\in M_{1\times mn}\to dm_{1}\in M_{m\times n}$ and $dm_{2}\in M_{1\times mn}\to dm_{2}\in M_{m\times n}$.

Step 3: The diffusion process is described as

(1) $C\left(lp,cp\right)=(S\left(lp,cp\right)+dm_{1}\left(lp,cp\right)+dm_{2}\left(lp,cp\right))\;\mathrm{mod}\;256$.

(2) $C\left(lp,i\right)=(S\left(lp,i\right)+dm_{1}\left(lp,i\right)+dm_{2}\left(lp,i\right)+C\left(lp,i-1\right))\;\mathrm{mod}\;256,i=cp+1,cp+2,cp+3,\ldots ,n$.

(3) $C\left(lp,i\right)=(S\left(lp,i\right)+dm_{1}\left(lp,i\right)+dm_{2}\left(lp,i\right)+C\left(lp,i+1\right))\;\mathrm{mod}\;256,i=cp-1,cp-2,cp-3,\ldots ,1$.

(4) $C\left(i,j\right)=S\left(i,j\right)⊕dm_{1}\left(i,j\right)⊕dm_{2}\left(i,j\right)⊕C\left(i+1,j\right),i=lp-1,lp-2,lp-3,\ldots ,1,j=1,2,3,\ldots ,n$.

(5) $C\left(i,j\right)=S\left(i,j\right)⊕dm_{1}\left(i,j\right)⊕dm_{2}\left(i,j\right)⊕C\left(i-1,j\right),i=lp+1,lp+2,lp+3,\ldots ,m,j=1,2,3,\ldots ,n$.

Output: *C* ($C\in M_{m\times n}$)

## 4. Performance Analysis

To evaluate the performance of the SFCF-IE algorithm, in this paper, we will analyze the encryption effect and security of the algorithm from the visualization, key space analysis, histogram analysis, key sensitivity, information entropy, correlation, and NIST.

### 4.1. Visualization

**Figure 5:**
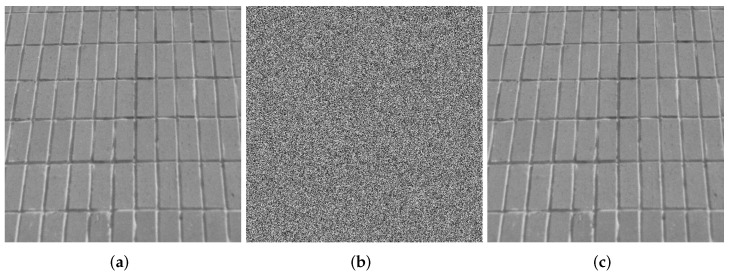
Visualization of SFCF-IE for image ‘1.5.01’ (512 × 512). (**a**) plaintext of ‘1.5.01’. (**b**) ciphertext of ‘1.5.01’. (**c**) decrypted ‘1.5.01’.

**Figure 6:**
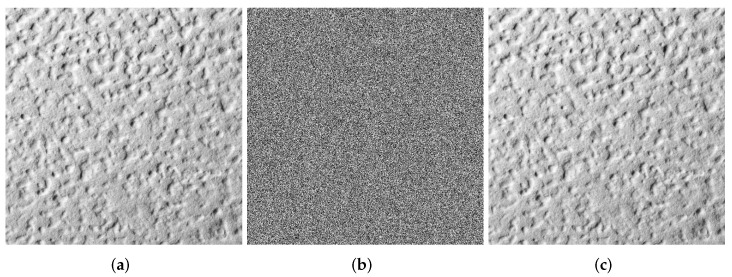
Visualization of SFCF-IE for image ‘1.5.03’ (512 × 512). (**a**) plaintext of ‘1.5.03’. (**b**) ciphertext of ‘1.5.03’. (**c**) decrypted ‘1.5.03’.

**Figure 7:**
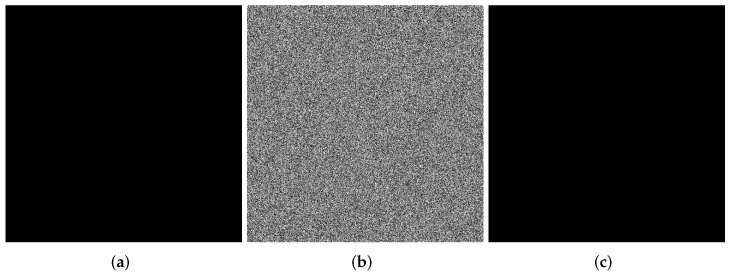
Visualization of SFCF-IE for image Black (512 × 512). (**a**) plaintext of Black. (**b**) ciphertext of Black. (**c**) decrypted Black.

Images are selected from the USC-SIPI Image Database for visualization analysis. The visual analysis of the SFCF-IE are shown in Figure 5, Figure 6 and Figure 7, including the encryption and decryption results of the image. The visualization results show that the ciphertext image obtained by the SFCF-IE is a noise image, and the attacker cannot obtain any information from the ciphertext image.

### 4.2. Key Analysis

The original key of SFCF-IE is generated by a hash function, and the rest of the keys are converted from the original key, so the key space of the SFCF-IE is $2^{256}$. The key space of the SFCF-IE is much larger than $2^{100}$, so the algorithm in this paper is sufficient to resist external exhaustive attacks.

**Figure 8:**
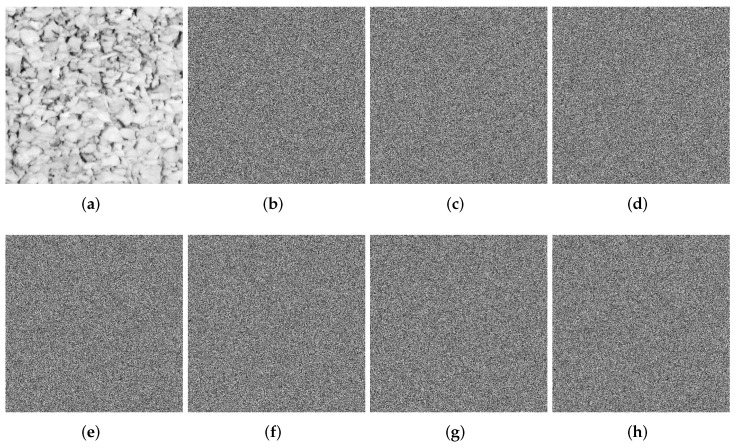
Key sensitivity analysis of SFCF-IE. (**a**) decrypted by *K*. (**b**) decrypted by $K_{1}$. (**c**) decrypted by $K_{2}$. (**d**) decrypted by $K_{3}$. (**e**) decrypted by $K_{4}$. (**f**) decrypted by $K_{5}$. (**g**) decrypted by $K_{6}$. (**h**) decrypted by $K_{7}$.

In addition to being large, the key of a cryptographic system also needs to be sensitive enough. A good cryptographic algorithm must be highly sensitive to small changes in the key. Figure 8 analyzes the key sensitivity of the SFCF-IE. The original key is *K*, and the slightly changed keys are $K_{1},K_{2},K_{3},K_{4},K_{5},K_{6}$. Decrypt with the correct key and the wrong key, respectively.

K=beb9b8c4ef16383cd3b1945b8dd8b2873f7bbbb29ee7a6903d29bd6c94095aa9

$K_{1}=beb9b8c4ef16383cd3b1945b8dd8b2873f7bbbb29ee7a6903d29bd6c94095aaa$

$K_{2}=beb9b8c4ef16383cd3b1945b8dd8b2873f7bbbb29ee7a6903d29bd6c94095aa8$

$K_{3}=aeb9b8c4ef16383cd3b1945b8dd8b2873f7bbbb29ee7a6903d29bd6c94095aa9$

$K_{4}=ceb9b8c4ef16383cd3b1945b8dd8b2873f7bbbb29ee7a6903d29bd6c94095aa9$

$K_{5}=beb9b8c4ef16383cd3b1945b8aa8b2873f7bbbb29ee7a6903d29bd6c94095aa9$

$K_{6}=beb9b8c4ef16383cd3b1945b8dd8b2873f7bbbb29ee7a6903d2abd6c94095aa9$

$K_{7}=beb9b8c4ef16483cd3b1945b8dd8b2873f7bbbb29ee7a6903d29bd6c94095aa9$

### 4.3. Histogram Analysis

**Figure 9:**
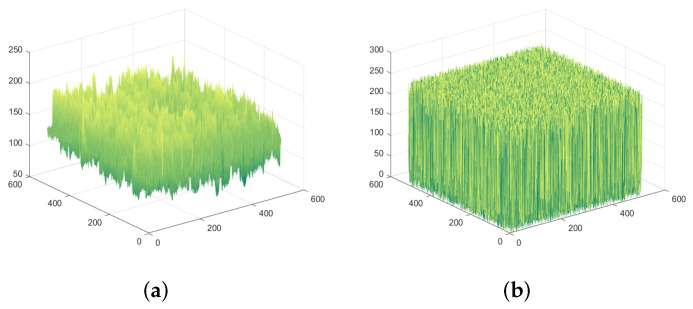
Spatiotemporal histogram of ‘1.5.01’. (**a**) Plaintext spatiotemporal histogram of ‘1.5.01’. (**b**) Ciphertext spatiotemporal histogram of ‘1.5.01’.

**Figure 10:**
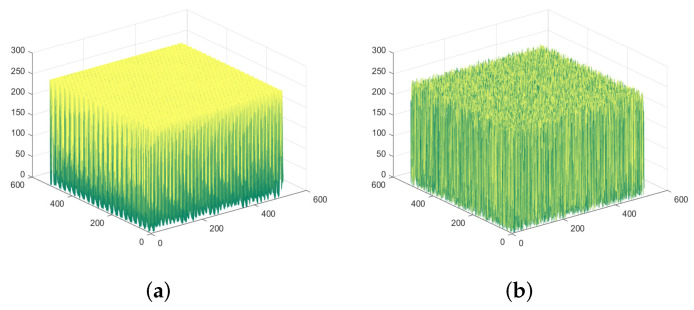
Spatiotemporal histogram of ‘1.5.02’. (**a**) Plaintext spatiotemporal histogram of ‘1.5.02’. (**b**) Ciphertext spatiotemporal histogram of ‘1.5.02’.

The histogram analysis can intuitively reflect the distribution characteristics of pixels. If the ciphertext cannot cover up the statistical characteristics of the image, the attacker will infer the information distribution of the plaintext according to the ciphertext to crack the algorithm. The histogram analysis of the SFCF-IE is shown in Figure 9 and Figure 10.

Experimental results show that the algorithm can well mask the statistical properties of plaintext images. After encryption, the gray value appears with approximately equal probability.

### 4.4. Information Entropy Analysis

Shannon’s theorem states that when the degree of disorder of information increases, its entropy value will increase, and when the probability of occurrence of each element in the information is equal, its entropy value will reach its maximum value. The calculation formula of information entropy is

$$H=\sum_{i=0}^{255}p(g_{i})\log_{2}\frac{1}{p(g_{i})}.$$

The information entropy of the SFCF-IE is shown in Table 2. In addition, the comparison with the average information entropy of some classical algorithms [34,35,36,37,38] are shown in Table 3.

The experimental results show that the ciphertext information entropy of the SFCF-IE is close to 8. Compared with other algorithms, the information entropy of the SFCF-IE is closer to the theoretical value, so it can be shown that the SFCF-IE has better encryption effect.

### 4.5. Correlation Analysis

The attacker can attack an image by analyzing the correlation between adjacent pixels of the image, so the encryption algorithm should eliminate this correlation. The adjacent pixel correlation is defined as,

$$r_{\rho }=\frac{\mathrm{cov}(x,y)}{\sqrt{D(x)\cdot D(y)}}.$$

**Figure 11:**
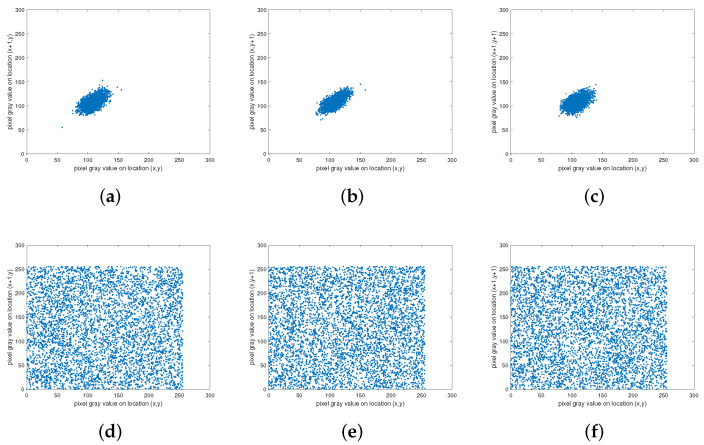
Correlation analysis of ‘1.5.06’. (**a**) Horizontal of plaintext. (**b**) Vertical of plaintext. (**c**) Diagonal of plaintext. (**d**) Horizontal of ciphertext. (**e**) Vertical of ciphertext. (**f**) Diagonal of ciphertext.

Figure 11 is the result of the SFCF-IE adjacent pixel correlation analysis. When the correlation between adjacent pixels is strong, the image presents a state of aggregation, and when the correlation between adjacent pixels is weak, the image presents a state of divergence.

Table 4 shows the quantitative analysis results of the correlation between adjacent pixels of the SFCF-IE, and the comparison results with some classical algorithms (Refs. [34,35,36,37,38]) are shown in Table 5.

### 4.6. NIST for Ciphertexts

Use the NIST to test whether the ciphertext image obtained by the SFCF-IE is random. The NIST test results are shown in Table 6. The plaintext image fails in 14 tests, and one test is successful. The plaintext image does not have randomness, and the distribution of pixel values has certain regularity. The ciphertext passed 15 tests, indicating that the ciphertext image has good randomness, and the attacker cannot find information related to the plaintext from the ciphertext image. Therefore, the SFCF-IE has strong security.

## 5. Conclusions

In this work, a 2D-SFCF hyperchaotic system is proposed. Through Attractor, Lyapunov exponent, and bifurcation graph, NIST verified that the 2D-SFCF has better dynamic behavior and larger parameter space than low-dimensional chaotic systems. In addition, the parameter space of the 2D-SFCF in a hyperchaotic state is continuous. Based on the 2D-SFCF, we propose a new image encryption algorithm called SFCF-IE. Through key analysis, information entropy analysis, correlation analysis, NIST, and other methods, it is verified that THE 2D-SFCF has high security and high practical value and is widely used in secure real-time communication of images and other occasions.

## Figures and Tables

**Table 1:** NISTtest of plaintext and ciphertext.

Statistical Test	a = 9.7, b = 8				a = 2.8, b = 10			
	X		Y		X		Y	
	*p*-Value	Result	*p*-Value	Result	*p*-Value	Result	*p*-Value	Result
Longest run of ones	0.419021	Success	0.096578	Success	0.236810	Success	0.534146	Success
Overlapping template matching	0.616305	Success	0.383827	Success	0.534146	Success	0.574903	Success
Random excursions variant	0.671779	Success	0.350485	Success	0.976060	Success	0.888137	Success
Rank	0.657933	Success	0.350485	Success	0.816537	Success	0.911413	Success
Frequency	0.066882	Success	0.350485	Success	0.494392	Success	0.108791	Success
Universal	0.657933	Success	0.494392	Success	0.350485	Success	0.883171	Success
Random excursions	0.602458	Success	0.275709	Success	0.862344	Success	0.995711	Success
Block frequency	0.289667	Success	0.911413	Success	0.006661	Success	0.779188	Success
Cumulative sums	0.191687	Success	0.289667	Success	0.574903	Success	0.236810	Success
Runs	0.213309	Success	0.816537	Success	0.816537	Success	0.085587	Success
Serial	0.779188	Success	0.616305	Success	0.883171	Success	0.289667	Success
Spectral	0.045675	Success	0.816537	Success	0.883171	Success	0.851383	Success
Approximate entropy	0.955835	Success	0.455937	Success	0.816537	Success	0.383827	Success
Nonoverlapping template matching	0.971699	Success	0.383827	Success	0.534146	Success	0.739918	Success
Linear complexity	0.574903	Success	0.534146	Success	0.455937	Success	0.455937	Success

**Table 2:** Information entropy of SFCF-IE.

Image	Plaintext	Ciphertext
1.4.01	6.3291	7.9998
1.4.02	7.1882	7.9998
1.4.03	6.6188	7.9998
1.4.04	6.1911	7.9998
1.4.05	7.1177	7.9998
1.5.01	5.6826	7.9993
1.5.02	5.8145	7.9993
1.5.03	6.9857	7.9993
1.5.04	6.4154	7.9993
1.5.05	6.8087	7.9993
1.5.06	5.1332	7.9993
1.5.07	6.7359	7.9993
Black	0	7.9993
White	0	7.9992
Average	5.5015	7.9995

**Table 3:** Information entropy comparison.

Algorithms	SFACF-IE	Ref. [34]	Ref. [35]	Ref. [36]	Ref. [37]	Ref. [38]
Information entropy	7.9995	7.993	7.9995	7.9993	7.9992	7.9972

**Table 4:** Correlation coefficients of SFCF-IE.

Image	Size	Plaintext			Ciphertext		
		Horizontal	Vertical	Diagonal	Horizontal	Vertical	Diagonal
1.4.01	1024 × 1024	0.9468	0.9172	0.9175	−0.0002	−0.0005	0.0011
1.4.02	1024×1024	0.9749	0.9347	0.9209	−0.0002	−0.00002	0.0005
1.4.03	1024×1024	0.9700	0.9557	0.9503	−0.0010	−0.0009	0.0005
1.4.04	1024×1024	0.9739	0.9691	0.9633	0.0006	0.0007	0.0006
1.4.05	1024×1024	0.9894	0.9734	0.9670	0.0012	0.0010	−0.0011
1.5.01	512×512	0.8383	0.8956	0.7945	−0.0003	−0.0053	−0.0009
1.5.02	512×512	0.8893	0.8940	0.8043	0.0009	0.0030	−0.0003
1.5.03	512×512	0.8821	0.9214	0.8119	0.0006	0.0019	−0.0004
1.5.04	512×512	0.7553	0.7146	0.5593	−0.0006	−0.0014	0.0001
1.5.05	512×512	0.9510	0.9582	0.9206	−0.0015	0.0039	−0.0006
1.5.06	512×512	0.6165	0.5006	0.4633	0.0020	0.0017	−0.0017
1.5.07	512×512	0.7163	0.8144	0.5950	0.0007	0.0003	−0.0024
Black	512×512	1	1	1	0.00003	−0.0022	0.0031
White	512×512	1	1	1	0.0012	−0.0019	−0.0016
Average		0.8931	0.8892	0.8334	0.0002	0.00005	−0.0002

**Table 5:** Correlation coefficients of SFCF-IE.

Algorithms	SFCF-IE	Ref. [34]	Ref. [35]	Ref. [36]	Ref. [37]	Ref. [38]
Horizontal	0.0002	0.0048	−0.0042	0.0022	−0.0519	−0.0016
Vertical	0.00005	−0.0025	−0.0049	0.0017	−0.0385	−0.0026
Diagonal	−0.0002	−0.0072	−0.0045	0.0019	0.0046	0.0116

**Table 6:** NISTtest of SFCF-IE.

Number	Statistical Test	Plaintext		Ciphertext	
		*p*-Value	Result	*p*-Value	Result
1	Longest run of ones	0	Fail	0.151616	Success
2	Overlapping template matching	0	Fail	0.611108	Success
3	Random excursions variant	0	Fail	0.949602	Success
4	Rank	0	Fail	0.016431	Success
5	Frequency	0	Fail	0.258961	Success
6	Universal	0	Fail	0.559523	Success
7	Random excursions	0	Fail	0.602458	Success
8	Block frequency	0	Fail	0.199580	Success
9	Cumulative sums	0	Fail	0.855534	Success
10	Runs	0	Fail	0.113706	Success
11	Serial	0	Fail	0.714660	Success
12	Spectral	0	Fail	0.509162	Success
13	Approximate entropy	0	Fail	0.258961	Success
14	Non-overlapping template matching	0	Fail	0.953553	Success
15	Linear complexity	0	Success	Fail	Success

## Data Availability

Not applicable.
